# Supplementary material for: Formation of Prenylated Chalcone Xanthohumol Cocrystals: Single Crystal X-ray Diffraction, Vibrational Spectroscopic Study Coupled with Multivariate Analysis
Source: Molecules. 2019 Nov 21;24(23):4245. doi: 10.3390/molecules24234245 (PMC6930654; doi:10.3390/molecules24234245)
Supplement: Supplementary file 1 [file molecules-24-04245-s001.pdf]

Supplementary Materials

Article

Formation of Prenylated Chalcone Xanthohumol Cocrystals: Single Crystal X-ray Diffraction, Vibrational Spectroscopic Study Coupled with Multivariate Analysis

Iwona Budziak [1], Marta Arczewska [2,*,†], Daniel M. Kamiński [3,*,†]

[1] Department of Chemistry, University of Life Sciences in Lublin, Akademicka 15, 20-950 Lublin, Poland; iwona.budziak@up.lublin.pl
[2] Department of Biophysics, University of Life Sciences in Lublin, Akademicka 13, 20-950 Lublin, Poland
[3] Department of General and Coordination Chemistry and Crystallography, Institute of Chemical Sciences, Maria Curie-Sklodowska University in Lublin, pl. Marii Curie-Skłodowskiej 2, 20-031 Lublin, Poland
* Correspondence: marta.arczewska@up.lublin.pl (M.A.); daniel.kaminski@umcs.pl (D.M.K.); Tel./Fax: +(48-81)445-69-05 (M.A.); Tel./Fax: +(48-81)537-56-44 (D.M.K.)
† The authors contributed equally to this paper.

Crystallographic information file

XN_AC, XN_NIC, XN_CF, XN_GA CIF files

Figure S1. d_{norm} mapped on Hirshfeld surface for the target cocrystals a) XN–NIC, b) XN–CF, c) single XN molecule with AC, d) single XN molecule with GA, e) asymmetric part of unit cell of XN–AC, and f) asymmetric part of unit cell of XN–GA. 3

Figure S2. Fingerprints plots of a) XN–NIC, b) XN–CF, c) XN–AC, and d) N–GA. 4

Figure S3. Related close contact contributions of intermolecular interactions to the Hirshfeld surface area of the target cocrystals. 5

Figure S4. FT-IR spectra for the coformers, the target cocrystals and physical mixtures. All spectra were normalized at the maximum intensity in the range between 1750–600 cm^{-1}. 6

Figure S5. Raman spectra of coformers, the target cocrystals and XN crystal. (a) XN–NIC, (b) XN–GA, (c) XN–AC and (d) XN–CF. All Raman spectra were normalized to the most intense band in the range 1750 – 600 cm^{-1}. 8

Table S2. Band assignments for the data the data acquired form Raman spectroscopy. 9

Figure S6. Scree plot obtained from PCA. Raman spectroscopy in the range 1700 – 1000 cm^{-1} (a), FTIR spectroscopy in the range 3700 – 2700 cm^{-1} (b), and 1800 – 1000 cm^{-1} (c). 10

Figure S7. PCA 2D score plots. PC1 and PC3 (a), PC2 and PC3 (b) calculated based on Raman spectroscopy data. 11

Figure S8. PC1, PC2 and PC3 correlation loading plots. 12

Figure S9. PCA 2D score plot: PC1 and PC3 (a), PC2 and PC3 (b) calculated based on the FT-IR data in the range 3700 – 2700 cm^{-1} (red symbols represent the physical mixtures, blue symbols – the cocrystals, black symbol –XN). 13

Figure S10. PCA 2D score plot: PC1 and PC3 (a), PC2 and PC3 (b) calculated based on the FT-IR data in the range 1800 – 1000 cm^{-1} (red symbols represent the physical mixtures, blue symbols – the cocrystals, black symbol –XN). 14

Figure S11. PC1, PC2 and PC3 correlation loading plots. The regions (a) 3700 – 2700 cm^{-1} and (b) 1800 – 1000 cm^{-1}. 15

Figure S12. X-ray powder diffraction patterns obtained after XN grinding with selected coformers (NIC, AC and GA). Based on the data it is difficult to conclude if the samples are cocrystals or a physical mixture of compounds. 16

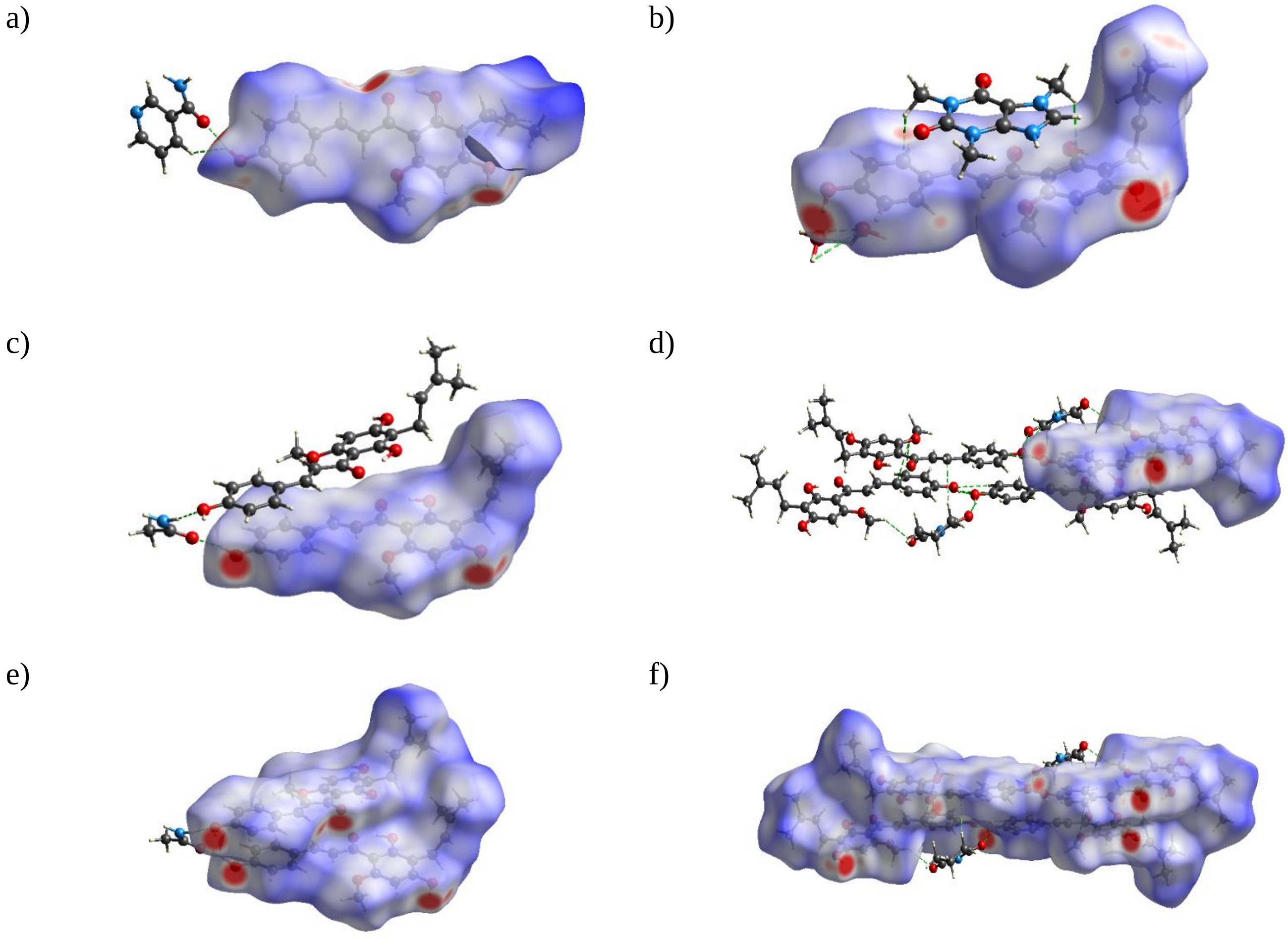

Figure S1. d_{norm} mapped on Hirshfeld surface for the target cocrystals (**a**) XN–NIC, (**b**) XN–CF, (**c**) single XN molecule with AC, (**d**) single XN molecule with GA, (**e**) asymmetric part of unit cell of XN–AC, and (**f**) asymmetric part of unit cell of XN–GA. The normalized distance d_{norm} to the nearest nuclei is marked and connected with strength of all types of the intermolecular contacts. The hydrogen bonds of the O···H contacts type are visible as the largest red spots. The red spots indicate the O···H bonds between the XN and the selected coformer molecules, and also the O···H interactions between the opposite XN molecules.

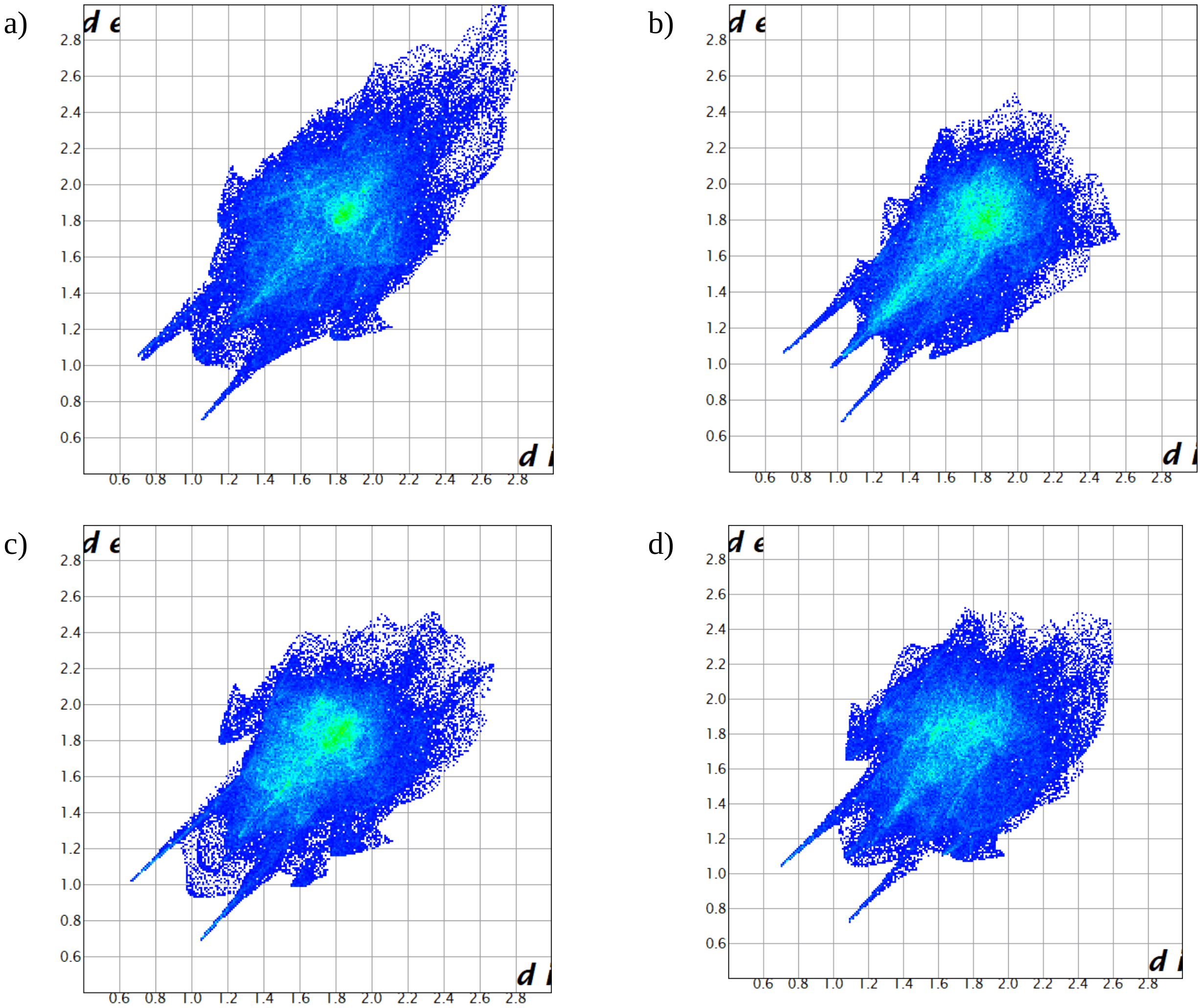

Figure S2. Fingerprints plots of (**a**) XN–NIC, (**b**) XN–CF, (**c**) XN–AC, and (**d**) XN–GA. The fingerprint plots show that the distributions of the nearby exterior (d_e) and interior (d_i) atom distances are very similar to the H···O and O···H interactions in all XN cocrystals. The significant contribution of polar interactions (O···H/H···O and N···H/H···N) is shown as long sharp spikes in all the cocrystals. The C···H/H···C show a symmetric pair of wings while the H···H contacts show asymmetric spots spread over a large area as broad peaks in all the cocrystals. The overall 2D fingerprint plots are similar to each other with subtle changes in the percentage contribution of these contacts.

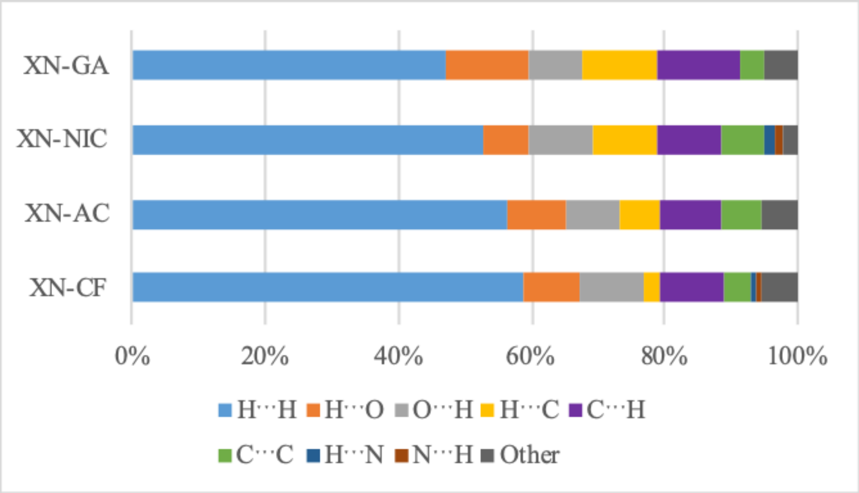

Figure S3. Related close contact contributions of intermolecular interactions to the Hirshfeld surface area of the target cocrystals. As can be seen, the structures are dominated by the weak H···H contact in the range from 47.1% to 58.7%. The contribution of the other contacts (O···H/H···O and N···H/H···N) is 8.1%–12.5% and 0.0%–1.4%. The contribution of the strongest hydrogen bonds O···H and H···O is almost the same in all structures while that of H···N and N···H bonds is minor.

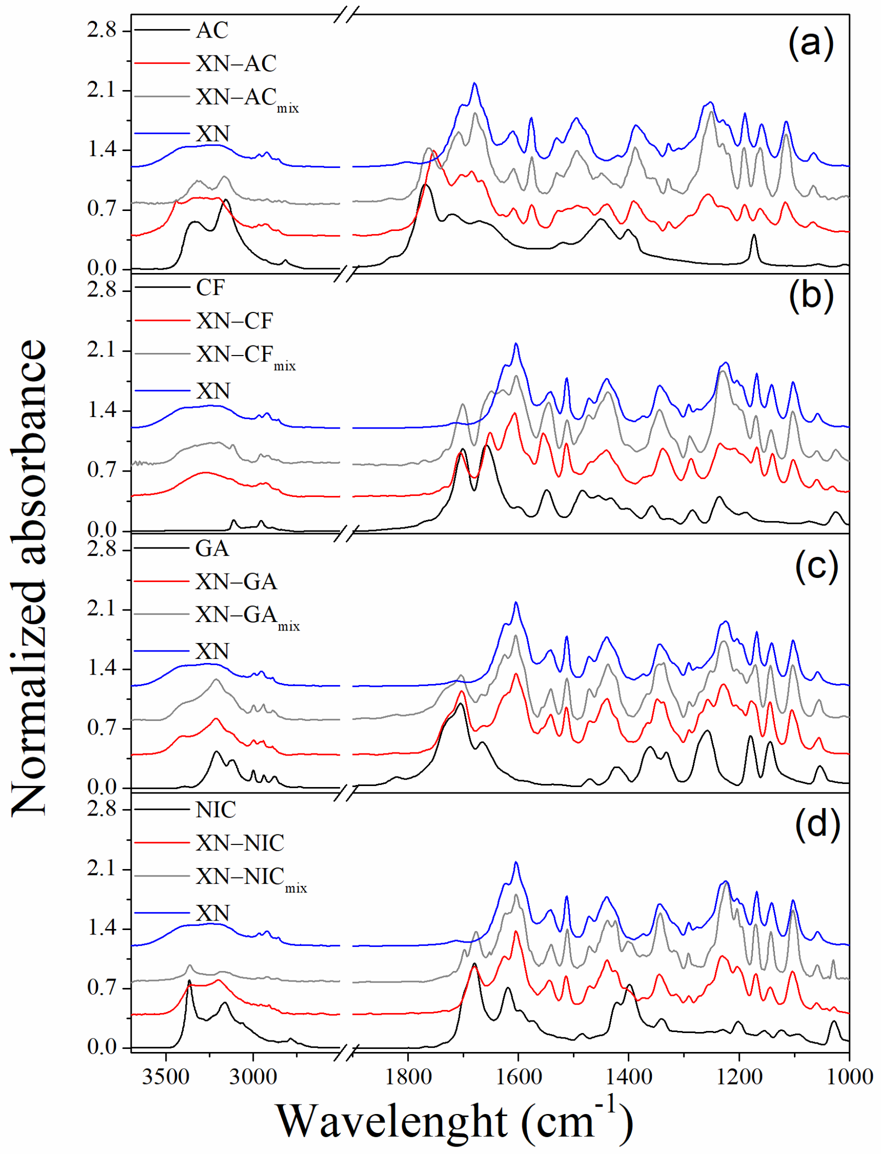

Figure S4. FTIR spectra for the coformers, the target cocrystals, and physical mixtures. All spectra were normalized at the maximum intensity in the range between 1750–600 cm^{-1}.

Table S1. Band assignment for the data acquired from the FTIR spectroscopy.

**Vibrational assignment**	**XN**	**XN–NIC**	**XN–GA**	**XN–CF**	**XN–AC**
ν(NH)					3440
	3390	3352	3389		
ν(OH), ν(CH) in	3189	3191	3192		3202
aromatic ring				3277	
$\nu_{as}(OCH_3)$	2963		2970	2928	2929
	2915	2908	2911		2856
$\nu_{s}(OCH_3)$	2855		2854		
			1730	1734	
			1704	1704	
Amide I		1680	1683	1651	1667
ν(C=O)	1626	1623	1627	1616	1625
ν(CC)	1604	1603	1604	1608	1608
					1593
Amide II, ν(C = N), *trans* –CH = CH–	1542	1545	1543	1554	1542
δ(CC)	1513	1512	1512	1512	1510
	1471	1471	1471	1471	1471
	1440	1439	1437	1442	1438
ν(CN), δ(NH)		1402			1391
	1343	1344	1348	1340	1345
ν(CC) in aromatic ring	1291	1292	1292	1287	1292
δ(OH)	1257	1257	1257		1257
	1224	1231	1227	1232	1227
	1170	1169	1175	1169	1170
ν(CC) in aromatic ring, ν(OH)	1142	1142	1143	1137	1142
	1101	1103	1103	1101	1104
Substituted benzene	1059	1061	1055	1057	1059

*ν—stretching mode, δ—bending in plane, s—symmetric vibrations, as—asymmetric vibrations.

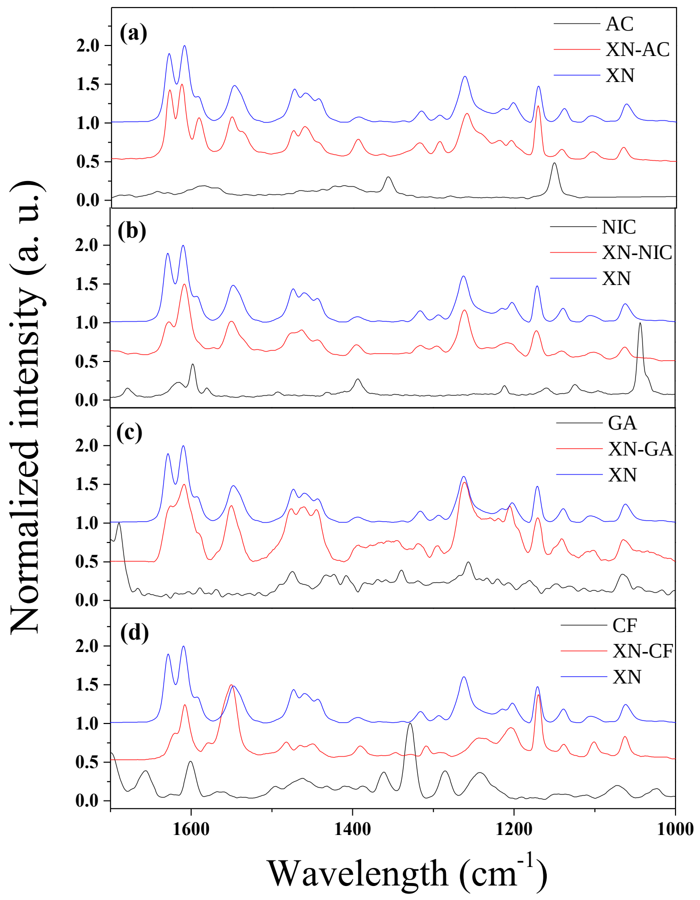

Figure S5. Raman spectra of coformers, the target cocrystals, and XN crystal. (**a**) XN–NIC, (**b**) XN–GA, (**c**) XN–AC, and (**d**) XN–CF. All Raman spectra were normalized to the most intense band in the range 1750–600 cm^{-1}.

Table S2. Band assignments for the data acquired from Raman spectroscopy.

Vibrational assignments	XN	XN–NIC	XN–GA	XN–AC	XN–CF
**ν(C = O)**	1628	1627	1624	1627	1620
**ν(C = C) in ring B, δ(NH) of NH_2**	1610	1607	1608	1612	1608
**ν(C = C) ring A**	1592	1584	1589	1592	1580
**δ(CH)**	1547	1547	1550	1550	1549
**ν(OCH_3) in ring A**					
				1538	
**ν(OCH_3) in ring A**	1473	1475	1476	1474	1482
**δ(CH_3)**	1458	1463	1461	1460	1464
	1442	1442	1444	1442	1449
**ν(CH_2), ν(CN) ν(CH_3), δ(CH_3)**	1392	1395	1395	1394	1390
**δ(CH = CH) in ring B**	1316	1317	1317	1318	1309
**δ(OH) in ring A**	1292	1296	1294	1293	1292
	1262	1261	1261	1260	1245
	1202	1204	1204	1205	1203
**δ(OH) in ring B**	1171	1171	1170	1171	1170
**δ(NH)**	1138	1139	1140	1143	1138
	1104	1102	1102	1104	1100
**δ(C–C)**	1061	1062	1062	1065	1062

*ν—stretching mode, δ—bending in-plane.

a)

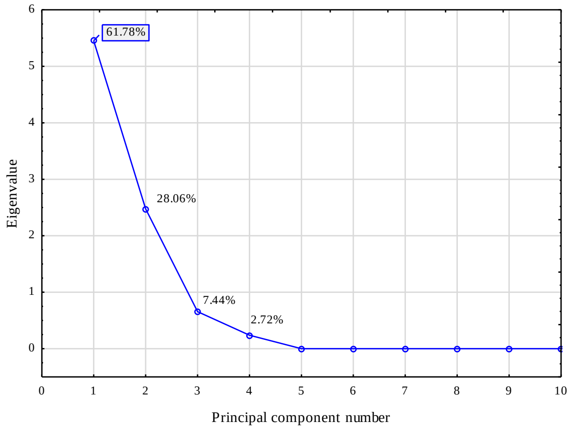

b)

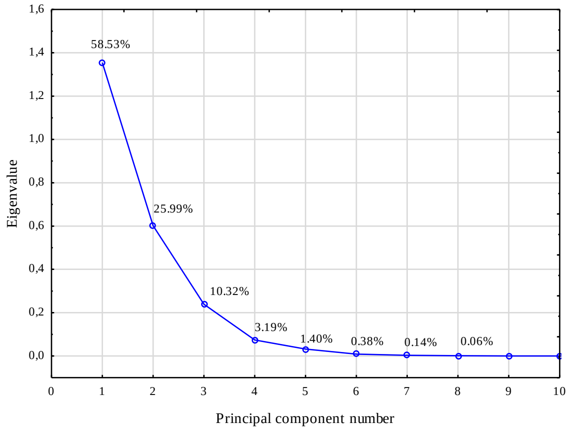

c)

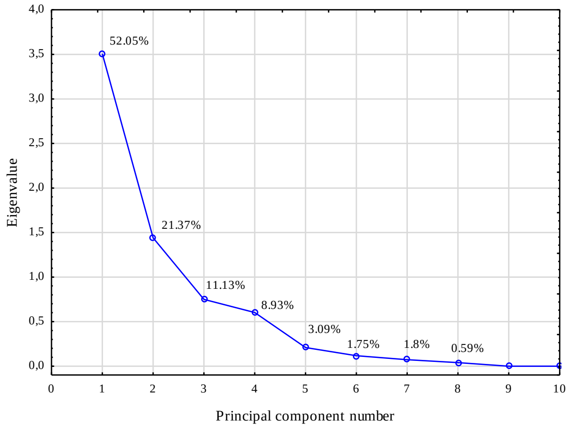

Figure S6. Scree plot obtained from PCA. (**a**) Raman spectroscopy in the range 1700–1000 cm^{-1}, (**b**) FTIR spectroscopy in the range 3700–2700 cm^{-1}, and (**c**) 1800–1000 cm^{-1}.

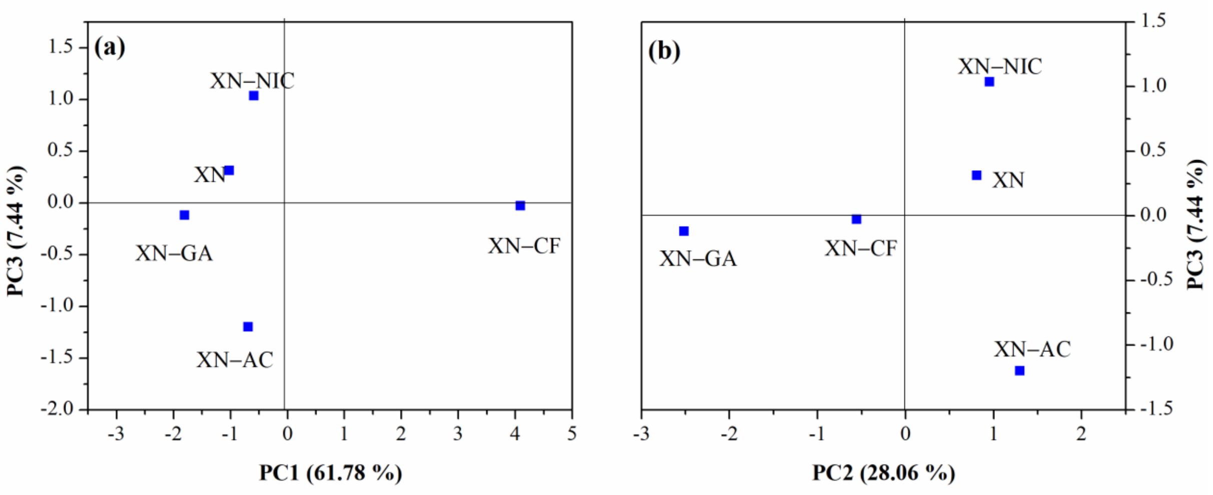

Figure S7. PCA 2D score plots: (**a**) PC1 and PC3 and (**b**) PC2 and PC3 calculated based on Raman spectroscopy data.

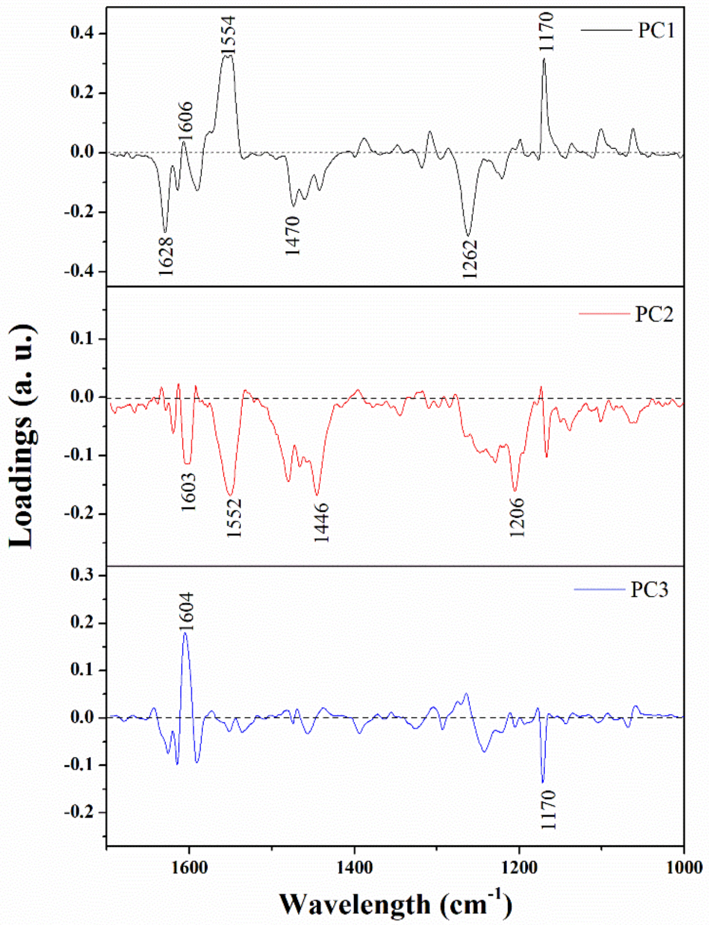

Figure S8. PC1, PC2, and PC3 correlation loading plots.

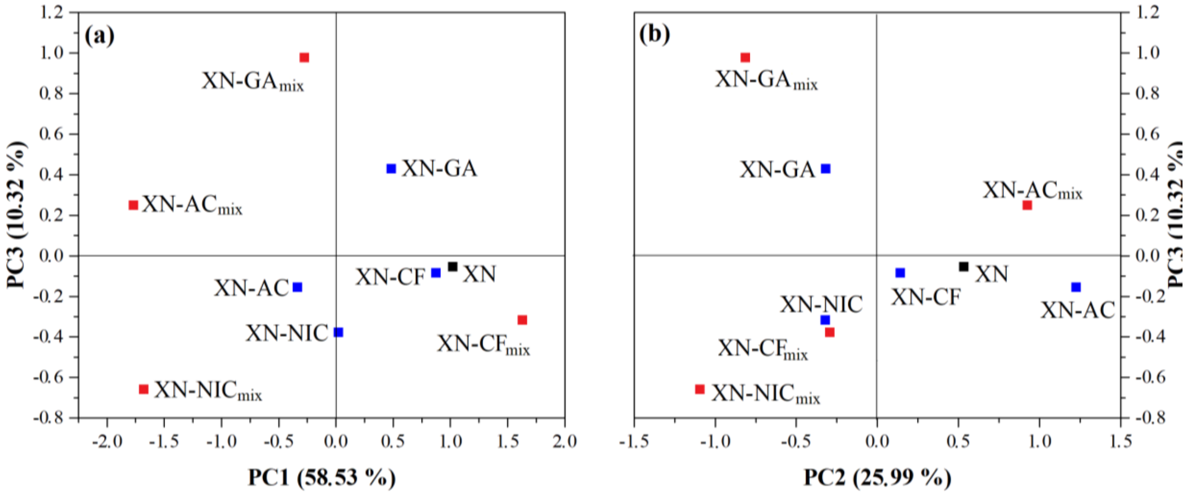

Figure S9. PCA 2D score plot: (**a**) PC1 and PC3 and (**b**) PC2 and PC3 calculated based on the FTIR data in the range 3700–2700 cm^{-1} (red symbols represent the physical mixtures, blue symbols represent the cocrystals, black symbols represent XN).

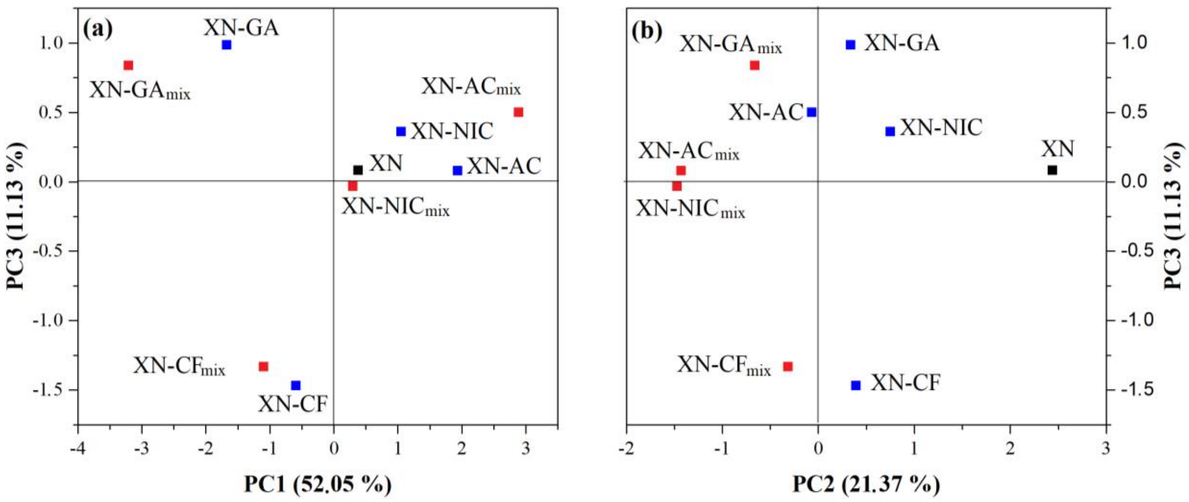

Figure S10. PCA 2D score plot: (**a**) PC1 and PC3 and (**b**) PC2 and PC3 calculated based on the FTIR data in the range 1800–1000 cm^{-1} (red symbols represent the physical mixtures, blue symbols represent the cocrystals, black symbols represent XN).

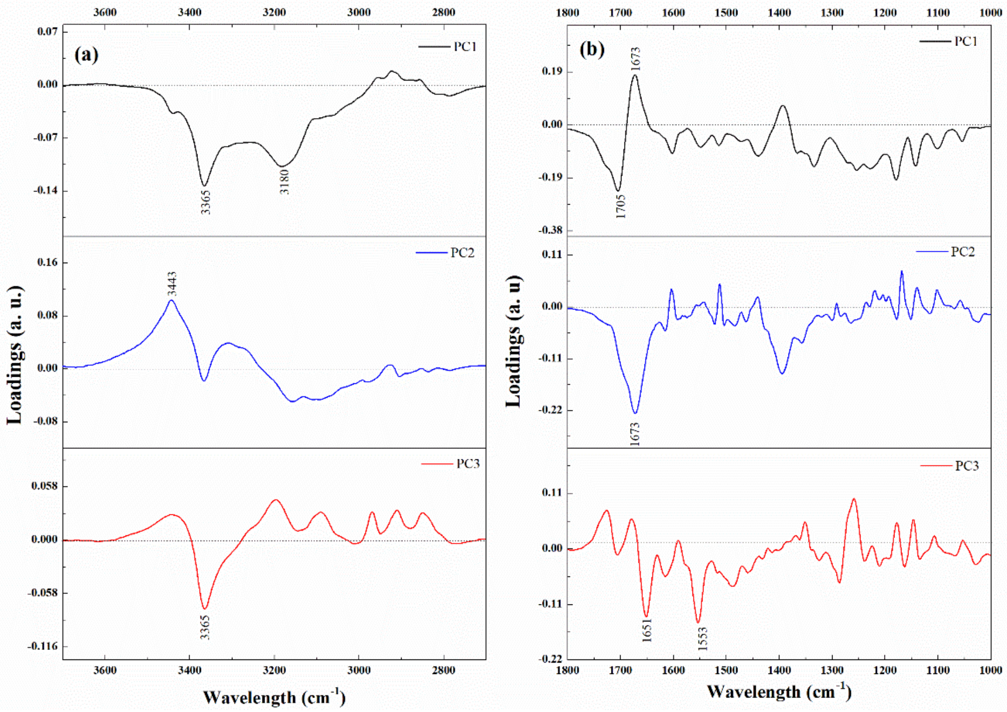

Figure S11. PC1, PC2, and PC3 correlation loading plots. The regions (**a**) 3700–2700 cm^{-1} and (**b**) 1800–1000 cm^{-1}.

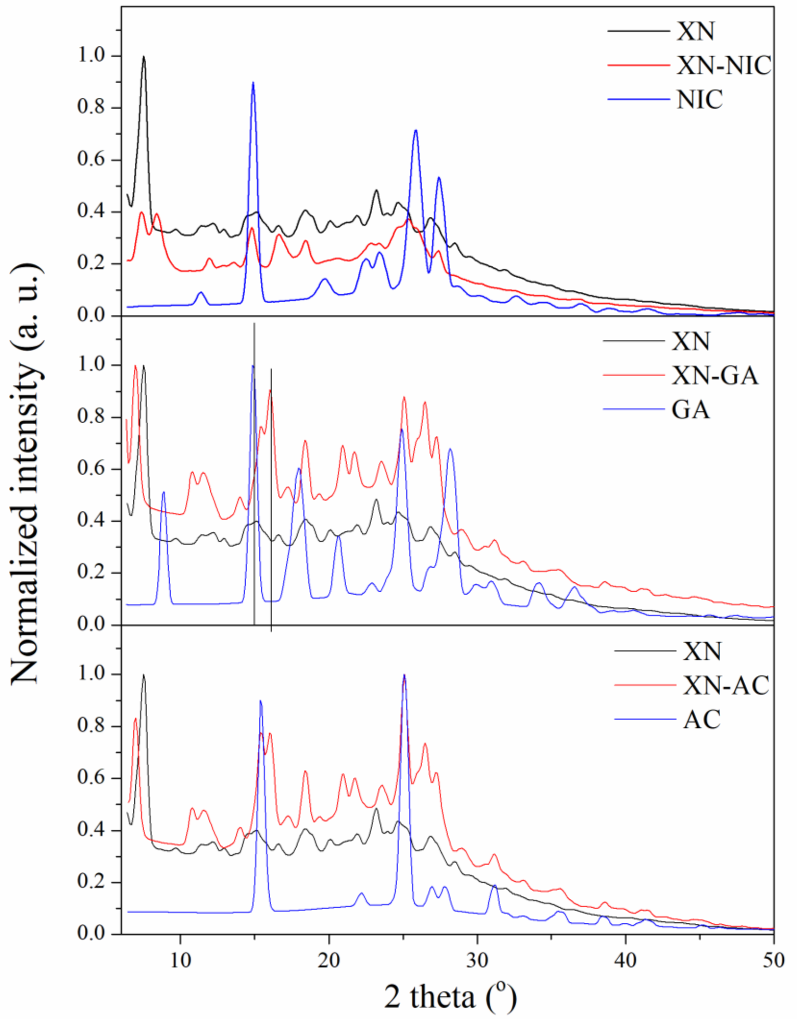

Figure S12. X-ray powder diffraction patterns obtained after XN grinding with selected coformers (NIC, AC, and GA). Based on the data it is difficult to conclude if the samples are cocrystals or a physical mixture of compounds.
